# Does the VHL polymorphisms rs779805 and rs1642742 affect renal cell carcinoma susceptibility, prognosis and survival in Central European population?

**DOI:** 10.1097/MD.0000000000036540

**Published:** 2023-12-15

**Authors:** Magdalena Chrabańska, Nikola Szweda-Gandor, Bogna Drozdzowska

**Affiliations:** a Department and Chair of Pathomorphology, Faculty of Medical Sciences in Zabrze, Medical University of Silesia, Katowice, Poland; b Department and Clinic of Internal Medicine, Diabetology and Nephrology, Medical University of Silesia, Zabrze, Poland.

**Keywords:** clear cell renal cell carcinoma, renal cell carcinoma, single nucleotide polymorphisms, VHL

## Abstract

Renal cell carcinoma (RCC) is a common malignant tumor of the urinary system. The von Hippel–Lindau (VHL) tumor suppressor gene play an essential role in the tumorigenic pathway of clear cell RCC (ccRCC). This study was aimed to clarify the influence of VHL polymorphisms on ccRCC susceptibility and survival in Central European population. We genotyped 2 single-nucleotide polymorphisms (SNPs) rs779805 and rs1642742 in VHL gene and assessed their associations with ccRCC risk, clinicopathologic parameters, and prognosis in 171 cases. The selected SNPs were genotyped by ROCHE LifeCycler 96 using tumor tissue-derived DNA. Both SNPs do not directly influence ccRCC susceptibility and overall survival. A significant associations were found between allele G and genotypes AG and GG of rs779805 in the VHL tumor suppressor gene and increased tumor size, as well as high nuclear grade. Furthermore, a statistically significant association was observed between rs1642742 of VHL gene and low pathological tumor stage and between rs779805 of VHL gene and high pathological tumor stage. Both investigated SNPs can be important prognostic indicators of RCC in the Central European population, because statistically significant associations were observed between evaluated VHL polymorphisms and the best known factors with proven prognostic significance in kidney cancer.

## 1. Introduction

Renal cell carcinoma (RCC) is the ninth most common cancer type worldwide.^[1,2]^ There are 3 main RCC subtypes that are determined by their histologic features: clear cell, papillary, and chromophobe RCC. Clear cell RCC (ccRCC) represents about 75% of all RCC cases, is the most aggressive form of this cancer type, and is known to be closely related with mutation of the von Hippel-Lindau gene (VHL). The VHL gene is a tumor suppressor gene of 639 coding nucleotides distributed over 3 exons and located at chromosome 3p25.3.^[2]^ VHL has been shown to be affected in more than 90% of the sporadic ccRCC cases, either by allelic deletion, promoter methylation (19%), or mutations (70%–80%).^[3–5]^ Given the multiple functions of the VHL gene product (pVHL) the inactivation of VHL is a critical point in the initiation of tumor development in the context of ccRCC.^[6–8]^ The VHL gene plays a significant role in tissue-specific responses to the oxygen accumulation and distribution.^[5]^ The pVHL is a crucial mediator of alpha (α) subunit of transcription factor hypoxia-inducible factor-1 (HIF-1). HIF-1 plays a key role in renal carcinogenesis through regulating genes involved in angiogenesis, erythropoiesis, cell adhesion, metastatic spread, apoptosis, and glucose metabolism. HIF-1α is the oxygen-regulated factor that determines HIF-1 activity. Under normal oxygen conditions, HIF-1α is targeted for ubiquitin-mediated degradation by the proteasome, which is mediated by pVHL acting as a component of the E3 ubiquitin–protein ligase complex. Under hypoxic conditions, degradation of HIF-1α is suppressed and then HIF-1α accumulates in the cells.^[9–11]^ Inactivation of pVHL leads to the HIF-1α overaccumulation, which facilitates oxygen delivery, adaptation to oxygen deprivation and angiogenesis. It subsequently leads to RCC development and progression. Therefore, genetic alterations in VHL gene may lead to enhanced cell survival and carcinogenesis. Some single-nucleotide polymorphisms (SNP) in VHL gene are highly frequent, but have a low penetrance. Generally, SNPs are responsible for different phenotypes and may modify cancer risk by influencing the gene function.^[12]^ However, previous studies have found conflicting proofs on the relationship between VHL SNPs and ccRCC susceptibility, with some studies indicating no association, while others indicate a positive association. Recently, we conducted a pilot study to evaluate selected VHL SNPs (rs779805 and rs1642742), and to identify their associations with the risk, survival and clinicopathologic parameters of ccRCC cases.^[13]^ In the contemporary study, we expanded upon our previous analysis, and reported findings from the entire case series. The integrated data can increase statistical power to investigate the role of 2 selected VHL SNPs (rs779805 and rs1642742) in ccRCC cases in Central European population. It should be mentioned that these selected SNPs were considered for the first time in this population in our research.

## 2. Materials and methods

### 2.1. Study population

The study included 171 tumors obtained from patients residing in Upper Silesia in Poland and diagnosed with sporadic renal cancer between January 2015 and January 2020. All tumors were histopathologically confirmed ccRCC. If another subtype of RCC was diagnosed, the tumor was excluded from the study. All submitted surgical specimens were complied with the guidance of the ISUP and the WHO for specimen handling, sampling and reporting.^[14,15]^ The tissue specimens were formalin-fixed and paraffin-embedded according to standard pathological tissue processing technique. Than 2 pathologists assessed hematoxylin and eosin stained slides according to WHO/ISUP grade and eighth edition of the American Joint Committee on Cancer (AJCC) TNM pathological staging category.^[16]^ Follow-up data included: day of surgical procedure, survival status, day of death, and/or day of last follow-up. The conventional study methodology was reported in accordance with “Strengthening the Reporting of Observational Studies in Epidemiology” guidelines.^[17]^

### 2.2. Control cases population

All information concerning control cases derived from the Ensembl database (origin: https://www.ensembl.org/index.html). Due to the fact that in this database there is no examined Polish population, we chose for comparison the European (northern and western) population, which is ethnically as close as possible to the study group.

### 2.3. Selection of gene and SNPs

Gene and SNPs related to renal carcinogenesis were selected through literature and bioinformatics database (http://www.ncbi.nlm.nih.gov/snp/) search. Consequently, 2 SNP loci in the gene VHL (rs1642742 and rs779805) were selected. MagCore Genomic DNA FFPE One-Step kit of the MagCore isolation system was used to carry out required genetic studies. Obtained material was then used for genetic tests (allelic discrimination of VHL polymorphisms). Conforming to probe manufacturer TaqMan Thermo Fisher Scientific following primers were used: VLH rs1642742 Context Sequence [VIC/FAM]GGACAGCTTGTATGTAAGGAGGTTT[A/G]TATAAGTAATTCAGTGGGAATTGCA and VLH rs779805 Context Sequence [VIC/FAM]GGCCTAGCCTCGCCTCCGTTACAAC[A/G]GCCTACGGTGCTGGAGGATCCTTCT (more information in Supplemental Digital Content, http://links.lww.com/MD/L11).

### 2.4. DNA extraction and genotyping

Genomic DNA was isolated using DNA isolation MagCore Genomic DNA FFPE One-Step Kit following the manufacturer instructions. The selected SNPs were genotyped by ROCHE LifeCycler 96. Real-time fluorescence-based quantitative polymerase chain reaction was conducted with the use of specific assays TaqMan genotyping method allelic discrimination. One blank control (MQ) was placed for marking each time in each 96-well detection unit as the quality control. Two genotypes for each tumor case were determined and their frequency was established.

### 2.5. Statistical analysis

Quantitative data included numbers and case percentage, mean and standard deviation (SD). SNP genotype and allele frequencies were tested against departure from Hardy-Weinberg equilibrium using Pearson Chi-square test. χ^2^ test with Yates correction was used to compare genotype frequencies between case and control populations. Survival time was calculated from the date of RCC diagnosis to the date of death or last follow-up. The survival times according to VHL polymorphisms were estimated using the Kaplan–Meier method. The *P* values and odds ratios were also calculated. A *P* value <.05 was considered statistically significant. All analysis were done using statistical software STATISTICA 13.1 (StatSoft Inc., Tulsa, OK, USA) and Microsoft Excel 2013 (Microsoft Corporation, Redmond, WA, USA).

### 2.6. Ethics statement

The Institutional Review Board of Medical University of Silesia opined that this study does not require the consent of the bioethics committee (PCN/CBN/0052/KB/243/22). The whole data were kept fully anonymous during the whole study.

## 3. Results

### 3.1. Subjects characteristics

The characteristics of the study subjects were shown in Table 1. The tumors were obtained form 67 (39.18%) women and 104 (60.82%) men with the mean age of 63.25 (SD = 11.1), ranging from 34 to 85 years. Size of the tumor ranged from 1 to 18 cm at the largest diameter. The mean length of follow-up was 66.7 months (SD = 10.8), with a median of 69.33 months. During follow-up period a death was recorded in 75 cases.

**Table 1:** Demographic and clinical characteristics of clear cell renal cell carcinoma cases.

Characteristics	
Number of tumor samples [n (%)]	171 (100%)
Age, yr [mean ± SD]	63.25 ± 11.10
Sex [n (%)]	
Female	67 (39.18%)
Male	104 (60.82%)
Type of surgical procedure [n (%)]	
Radical nephrectomy	105 (61.40%)
Partial nephrectomy	66 (38.60%)
Kidney tumor location [n (%)]	
Right side	87 (50.88%)
Left side	84 (49.12%)
Tumor size, cm (mean ± SD)	5.64 ± 1.98
Tumor stage [n (%)]	
pT1	101 (59.07%)
pT2	15 (8.77%)
pT3	54 (31.58%)
pT4	1 (0.58%)
WHO/ISUP grade [n (%)]	
G1	63 (36.84%)
G2	73 (42.69%)
G3	22 (12.86%)
G4	13 (7.61%)
Dead [n (%)]	75 (43.10%)

SD = standard deviation.

### 3.2. Comparison of genotype and allele distributions of VHL polymorphisms in the ccRCC cases and controls

Table 2 shows the genotypic and allelic distributions of the 2 tested VHL polymorphisms in ccRCC cases and controls. The selected SNPs in both control and cases adhered to the Hardy-Weinberg equilibrium. Similar frequencies in the distribution of rs1642742 A/G and rs779805 A/G polymorphisms of VHL gene were found between healthy controls and RCC cases for genotypic and allelic frequencies. This suggests that these polymorphisms do not influence ccRCC susceptibility.

**Table 2:** Allelic and genotypic frequencies of selected SNPs in the clear cell renal cell carcinoma cases and controls.

	Case population [n (%)]	Database control population [n (%)]	OR
VHL rs1642742			
AA	84 (49.12%)	43 (43.4%)	1.95
AG	32 (18.71%)	48 (48.5%)	0.66
GG	55 (32.16%)	8 (8.1%)	6.87
A	200 (58.48%)	134 (68.0%)	1.49
G	142 (41.52%)	64 (32.0%)	2.21
VHL rs779805			
AA	83 (48.54%)	43 (43.4%)	1.93
AG	77 (45.03%)	48 (48.5%)	1.60
GG	11 (6.43%)	8 (8.1%)	1.37
A	153 (60.71%)	134 (68.0%)	1.06
G	99 (39.29%)	64 (32.0%)	1.54

OR = odds ratio, SNP = single-nucleotide polymorphism, VHL = von Hippel–Lindau.

### 3.3. Associations between VHL polymorphisms and the clinicopathological features of ccRCC patients

In this study we assessed the effects of VHL polymorphisms on patients’ gender and age, tumor size, WHO/ISUP grade, and AJCC TNM pathologic primary tumor stage (pT).

No significant associations between the evaluated SNPs and patients’ gender and age were observed (Tables 3 and 4).

**Table 3:** Associations of the VHL polymorphisms with patients’ gender.

Genotypes of rs1642742	AA [n (%)]	AG [n (%)]	GG [n (%)]	*P*	Alleles of rs1642742	A [n (%)]	G [n (%)]	*P*
Females	37 (21.64%)	12 (7.02%)	18 (10.53%)	*P* = .78	Females	86 (25.15%)	48 (14.04%)	*P* > .99
Males	47 (27.49%)	20 (11.70%)	37 (21.64%)		Males	114 (33.33%)	94 (27.49%)	
Genotypes of rs779805	AA [n (%)]	AG [n (%)]	GG [n (%)]	*P*	Alleles of rs779805	A [n (%)]	G [n (%)]	*P*
Females	37 (21.64%)	27 (15.79%)	3 (1.75%)	*P* = .80	Females	101 (29.53%)	33 (9.65%)	*P* = .88
Males	46 (26.90%)	50 (29.24%)	8 (4.68%)		Males	142 (41.52%)	66 (19.30%)	

VHL = von Hippel–Lindau.

**Table 4:** Associations of the VHL polymorphisms with patients’ age.

Genotypes of rs1642742	AA [n (%)]	AG [n (%)]	GG [n (%)]	*P*	Alleles of rs1642742	A [n (%)]	G [n (%)]	*P*
30–40 yr	3 (1.75%)	2 (1.17%)	2 (1.17%)	*P* > .99	30–40 yr	8 (2.34%)	6 (1.75%)	*P* > .99
41–50 yr	12 (7.02%)	1 (0.58%)	8 (4.68%)	*P* = .40	41–50 yr	25 (7.31%)	17 (4.97%)	*P* > .99
51–60 yr	19 (11.11%)	4 (2.34%)	12 (7.02%)	*P* = .71	51–60 yr	42 (12.28%)	28 (8.19%)	*P* > .99
61–70 yr	31 (18.13%)	16 (9.36%)	18 (10.53%)	*P* = .39	61–70 yr	78 (22.81%)	52 (15.20%)	*P* = .28
71–80 yr	18 (10.53%)	7 (4.09%)	12 (7.02%)	*P* = .47	71–80 yr	43 (12.57%)	31 (9.06%)	*P* = .42
81–90 yr	1 (0.58%)	2 (1.17%)	3 (1.75%)	*P* = .12	81–90 yr	4 (1.17%)	8 (2.34%)	*P* > .99
Genotypes of rs779805	AA [n (%)]	AG [n (%)]	GG [n (%)]	*P*	Alleles ofrs779805	A [n (%)]	G [n (%)]	*P*
30–40 yr	3 (1.75%)	4 (2.34%)	0 (0.00%)	*P* = .84	30–40 yr	10 (2.92%)	4 (1.17%)	*P* > .99
41–50 yr	12 (7.02%)	9 (5.26%)	4 (2.34%)	*P* > .99	41–50 yr	33 (9.65%)	17 (4.97%)	*P* = .47
51–60 yr	18 (10.53%)	13 (7.60%)	3 (1.75%)	*P* = .65	51–60 yr	49 (14.33%)	19 (5.56%)	*P* = .39
61–70 yr	32 (18.71%)	30 (17.54%)	3 (1.75%)	*P* = .52	61–70 yr	94 (27.49%)	36 (10.53%)	*P* = .27
71–80 yr	17 (9.94%)	17 (9.94%)	1 (0.58%)	*P* = .94	71–80 yr	51 (14.91%)	19 (5.56%)	*P* = .10
81–90 yr	1 (0.58%)	4 (2.34%)	0 (0.00%)	*P* > .99	81–90 yr	6 (1.75%)	4 (1.17%)	*P* > .99

VHL = von Hippel–Lindau.

A significant associations were found between allele G and genotypes AG and GG of rs779805 in the VHL tumor suppressor gene and increased (>7 cm) tumor diameter. The associations of VHL polymorphisms with largest diameter of tumor were shown in Table 5.

**Table 5:** Associations of the VHL polymorphisms with tumor size.

Genotypes of rs1642742	AA [n (%)]	AG [n (%)]	GG [n (%)]	*P*	Alleles of rs1642742	A [n (%)]	G [n (%)]	*P*
≤4 cm	41 (23.98%)	12 (7.02%)	15 (8.77%)	*P* = .24	≤4 cm	94 (27.49%)	42 (12.28%)	*P* = .32
4–7 cm	26 (15.20%)	13 (7.60%)	19 (11.11)	*P* = .27	4–7 cm	65 (19.01%)	51 (14.91%)	*P* > .99
7–10 cm	13 (7.60%)	7 (4.09%)	14 (8.19%)	*P* > .99	7–10 cm	33 (9.65%)	35 (10.23%)	*P* > .99
>10 cm	4 (2.34%)	0 (0.00%)	7 (4.09%)	*P* = .94	>10 cm	8 (2.34%)	14 (4.09%)	*P* > .99
*P*	*P* = .09	*P* = .036	*P* = .084		*P*	*P* = .087	*P* = .79	
Genotypes of rs779805	AA [n (%)]	AG [n (%)]	GG [n (%)]	*P*	Alleles ofrs779805	A [n (%)]	G [n (%)]	*P*
≤4 cm	41 (23.98%)	25 (14.62%)	2 (1.17%)	*P* = .078	≤4 cm	107 (31.29%)	29 (8.48%)	*P* = .22
4–7 cm	25 (14.62%)	26 (15.20%)	7 (4.09%)	*P* = .12	4–7 cm	76 (22.22%)	40 (11.70%)	*P* = .30
7–10 cm	13 (7.60%)	20 (11.70%)	1 (0.58%)	***P* = .048**	7–10 cm	46 (13.45%)	22 (6.43%)	*P* > .99
>10 cm	4 (2.34%)	6 (3.51%)	1 (0.58%)	***P* = .049**	>10 cm	14 (4.09%)	8 (2.34%)	*P* > .99
*P*	*P* = .078	***P* = .047**	***P* = .048**		*P*	*P* > .99	***P* = .049**	

VHL = von Hippel–Lindau.

Moreover our analysis showed a statistically significant association between allele G and genotypes AG and GG of rs779805 in the VHL tumor suppressor gene and high (G3 and G4) nuclear grade. The associations of VHL polymorphisms with tumor nuclear grade (G) were shown in Table 6.

**Table 6:** Associations of the VHL polymorphisms with tumor nuclear grade (G).

Genotypes of rs1642742	AA [n (%)]	AG [n (%)]	GG [n (%)]	*P*	Alleles of rs1642742	A [n (%)]	G [n (%)]	*P*
G1	32 (18.71%)	16 (9.36%)	15 (8.77%)	*P* = .35	G1	80 (23.39%)	46 (13.45%)	*P* > .99
G2	38 (22.22%)	11 (6.43%)	24 (14.04%)	*P* = .44	G2	87 (25.44%)	59 (17.25%)	*P* = .089
G3	11 (6.43%)	5 (2.92%)	6 (3.51%)	*P* = .60	G3	27 (7.89%)	17 (4.97%)	*P* > .99
G4	3 (1.75%)	0 (0.00%)	10 (5.85%)	*P* = .98	G4	6 (1.75%)	20 (5.85%)	*P* > .99
*P*	*P* = .085	*P* = .074	*P* = .088		*P*	*P* > .99	*P* > .99	
Genotypes of rs779805	AA [n (%)]	AG [n (%)]	GG [n (%)]	*P*	Alleles of rs779805	A [n (%)]	G [n (%)]	*P*
G1	32 (18.71%)	28 (16.37%)	3 (1,75%)	*P* = .069	G1	92 (26.90%)	34 (9.94%)	*P* = .79
G2	38 (22.22%)	30 (17.54%)	5 (2,92%)	*P* = .071	G2	106 (30.99%)	40 (11.70%)	*P* = .29
G3	10 (5.85%)	11 (6.43%)	1 (0,58%)	***P* = .047**	G3	31 (9.06%)	13 (3.80%)	*P* = .097
G4	3 (1.75%)	8 (4.68%)	2 (1,17%)	***P* = .049**	G4	14 (4.09%)	12 (3.51%)	*P* > .99
*P*	*P* = .057	***P* = .049**	***P* = .048**		*P*	*P* = .084	***P* = .048**	

VHL = von Hippel–Lindau.

Finally, we evaluated the associations of the VHL polymorphisms with AJCC TNM pathologic stage of the primary tumor. Our analysis revealed a statistically significant association between rs1642742 of VHL gene and ccRCC diagnosed at low stage (pT1). On the contrary, a statistically significant association was found between rs779805 of VHL gene and high stage (pT3) tumors. The associations of VHL polymorphisms with pathologic stage of the primary tumor were shown in Table 7.

**Table 7:** Associations of the VHL polymorphisms with TNM pathologic stage of the primary tumor (pT).

Genotypes of rs1642742	AA [n (%)]	AG [n (%)]	GG [n (%)]	*P*	Alleles of rs1642742	A [n (%)]	G [n (%)]	*P*
pT1	53 (30.99%)	22 (12.87%)	26 (15.20%)	***P* = .049**	pT1	128 (37.43%)	74 (21.64%)	*P* > .99
pT2	7 (4.09%)	1 (0.58%)	7 (4.09%)	*P* = .33	pT2	15 (4.39%)	15 (4.39%)	*P* > .99
pT3	24 (14.04%)	9 (5.26%)	21 (12.28%)	*P* = .098	pT3	57 (16.67%)	51 (14.91%)	*P* > .99
pT4	0 (0.00%)	0 (0.00%)	1 (0.58%)	*P* = .087	pT4	0 (0.00%)	2 (0.58%)	*P* > .99
*P*	*P* = .08	*P* = .084	*P* = .066		*P*	*P* = .14	*P* = .22	
Genotypes of rs779805	AA [n (%)]	AG [n (%)]	GG [n (%)]	*P*	Alleles ofrs779805	A [n (%)]	G [n (%)]	*P*
pT1	52 (30.41%)	43 (25.15%)	6 (3.51%)	*P* = .074	pT1	147 (42.98%)	55 (16.08%)	*P* = .51
pT2	7 (4.09%)	6 (3.51%)	2 (1.17%)	*P* = .20	pT2	20 (5.85%)	10 (2.92%)	*P* > .99
pT3	24 (14.04%)	27 (15.79%)	3 (1.75%)	***P* = .048**	pT3	75 (21.93%)	33 (9.65%)	*P* = .31
pT4	0 (0.00%)	0 (0.00%)	1 (0.58%)	*P* > .99	pT4	0 (0.00%)	2 (0.58%)	*P* > .99
*P*	*P* = .052	*P* = .064	*P* = .067		*P*	*P* = .19	*P* = .28	

VHL = von Hippel–Lindau.

### 3.4. Effects on VHL polymorphisms on ccRCC patients survival

**Figure 1.**
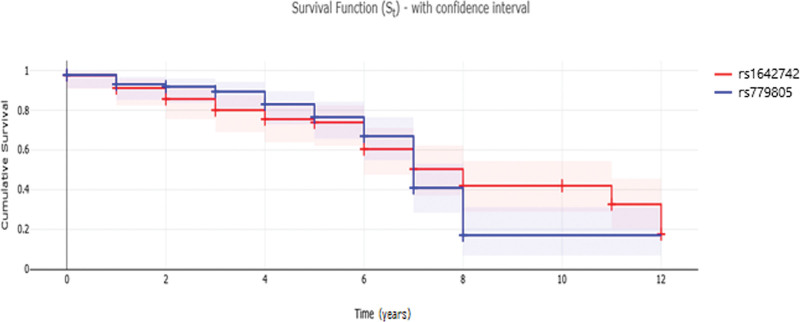
Kaplan–Meier survival curves illustrate clear cell renal cell carcinoma overall survival according to VHL polymorphisms. VHL = von Hippel–Lindau.

Cox regression analysis was used to access the associations between alleles and genotypes of evaluated SNPs in VHL and overall survival of all 171 cases with ccRCC. We did not observe a significant association of each allele or genotype of these SNPs with overall survival (Table 8). Kaplan–Meier curves were calculated for overall survival stratified according to VHL polymorphisms (Fig. 1).

**Table 8:** VHL polymorphisms and overall survival.

	Deaths [n (%)]	*P*	5-yr survival [n (%)]	*P*
VHL rs1642742				
AA	38 (50.67%)	*P* > .99	54 (50.47%)	*P* > .99
AG	11 (14.67%)		22 (20.56%)	
GG	26 (34.67%)		31 (28.97%)	
A	87 (58.00%)	*P* > .99	130 (60.75%)	*P* > .99
G	63 (42.00%)		84 (39.25%)	
VHL rs779805				
AA	36 (48.00%)	*P* = .57	55 (51.40%)	*P* = .69
AG	31 (41.33%)		48 (44.86%)	
GG	8 (10.67%)		4 (3.74%)	
A	103 (68.67%)	*P* > .99	158 (73.83%)	*P* = .73
G	47 (31.33%)		56 (26.17%)	

VHL = von Hippel–Lindau.

## 4. Discussion

SNPs are one of the most common types of genetic variations in the human genome. Most SNPs are “silent” and do not alter the function or expression of a gene. However SNPs in genes regulating DNA mismatch repair, cell cycle regulation, metabolism and immunity may be associated with genetic susceptibility to cancer. SNPs are located in different regions of genes such as promoters, exons, introns, as well as 5′- and 3′ untranslated regions (UTRs). Therefore, alterations in gene expression and their effect on cancer susceptibility vary depending on the location of the SNPs.^[18,19]^ For example, because the promoter region regulates the initiation and rate of gene transcription, promoter-related polymorphisms affect transcription factor binding that alter promoter activity, gene transcription, mRNA stability and translation. Then, these effects alter protein levels that potentially determine the individual susceptibility to diseases including cancer. Furthermore, polymorphisms in the promoter regions also affect neoplasm susceptibility by altering epigenetic mechanisms such as DNA methylation or histone modifications. In turn, the 3′-UTRs regulate gene expression through mRNA degradation and translation. It also controls polyadenylation, subcellular localization, translation efficiency, and mRNA degradation, as well as determines the fate of specific mRNAs and cell type-specific mRNA expression. Therefore, alternations in the 3′-UTR are involved in many neoplasms because they affect gene progression.^[19]^

In literature 2 SNPs, rs779805 and rs1642742 involving both A and G, located in the promoter and 3′-UTRs of the VHL gene are suspected to be informative and implicated in the occurrence of ccRCC worldwide.^[9,20–23]^ Because previous studies have found contradictory proofs on the relationship between VHL polymorphisms and ccRCC susceptibility, in the present study, we investigated the associations of VHL rs779805 and rs1642742 polymorphisms with risk, clinicopathological characteristics, and survival of ccRCC cases. We broadened our previous study,^[13]^ and reported findings from the entire case series.

Lv et al^[24]^ in the analysis of VHL rs779805 polymorphism found that genotype GG and allele G appear to be a risk factor for RCC development. Also van de Pol et al^[12]^ in multivariable-adjusted analyses found that individuals carrying the AG (vs AA) genotype of VHL rs779805 had a statistically significantly increased RCC and ccRCC risk, and the GG (vs AA) genotype was associated with a statistically significantly increased RCC, especially ccRCC, risk. Wang et al^[20]^ also confirmed that the existence of G allele or AG heterozygote in both rs779805 and rs1642742 in the VHL gene is of importance in RCC tumorigenesis. Moreover they observed lack of GG homozygote in the RCC patients, which suggest that the G variant is a lethal genetic mutation.^[20,23]^ Our study did not prove this hypothesis, because in our RCC population GG homozygotes were present in both VHL rs779805 and rs1642742. Additionally, our results are consistent with those of Qin et al^[9]^ who observed no significant differences in genotype and allele frequencies of VHL rs779805 polymorphisms between RCC patients and controls. Likewise, Bensouilah et al^[25]^ found similar frequencies in the distribution of rs1642742 and rs779805 polymorphisms of VHL between healthy controls and RCC patients for genotypic and allelic frequencies, which suggest that these VHL polymorphisms are not associated with the onset of RCC.

Some researchers also investigated effects of VHL polymorphisms on clinicopathological characteristics of RCC patients. Contrary to our observations, Qin et al^[9]^ found no significant associations between the genotype of rs779805 in VHL and pT, tumor size and tumor grade. Only in combined analysis, patients with ≥ 2 variant alleles of all 4 polymorphisms in VHL and HIF1A genes (VHL rs779805, HIF1A rs11549465, HIF1A rs11549467, HIF1A rs2057482) in their study were significantly associated with localized clinical stage and less frequency of lymph node metastasis. In turn, Wang et al^[20]^ found in multivariate analysis that age was an important factor in the genotype distributions of both rs779805 and rs1642742 in the VHL tumor suppressor gene. Their data suggests that the frequencies of AG heterozygote at both rs779805 and rs1642742 are much higher for late onset (≥50 years) RCC cases. Moreover AG heterozygote at rs1642742 was significantly associated with female gender in their research. Our study however did not prove these findings.

Recently, just a few studies have stated that VHL polymorphisms may have effects on the RCC prognosis, but with indecisive results. It has been reported that the most useful role for VHL mutational status may be in combination with other potential biomarkers, such as, for example, HIF1A or VEGF polymorphisms.^[4,26–29]^ In line with our results, Qin et al^[9]^ observed no significant associations between the genotypes of rs779805 in VHL and survival of RCC patients. However they suggested that variant alleles (≥1 vs 0) of the polymorphisms in VHL and HIF1A genes (VHL rs779805, HIF1A rs11549465, HIF1A rs11549467, HIF1A rs2057482) were an independent risk factor for patients’ survival.

All the discrepancies in the studies mentioned above might depend on different ethnicity of the studied populations. For example, Wang et al^[20]^ have found that the G allelic frequencies in rs779805 and rs1642742 of VHL gene in healthy patients from Taiwan are much lower than in the European population. It should be noted that most studies investigated the associations of VHL rs779805 and rs1642742 polymorphisms with risk, clinicopathological characteristics, and survival of ccRCC cases were conducted on Asian populations.^[9,20,24]^ To our knowledge, so far only one study has been conducted on the European (exactly Dutch) population.^[12]^ Therefore, more extensive research is required to address the clinical adequacy of these 2 genetic polymorphisms and RCC in European population.

## 5. Conclusions

In summary, we found evidence that both rs779805 and rs1642742 of VHL gene do not directly influence ccRCC susceptibility and overall survival in Upper Silesian population. However a significant associations were found between allele G and genotypes AG and GG of rs779805 in the VHL tumor suppressor gene and increased tumor size, as well as high nuclear grade. Moreover we observed a statistically significant association between rs1642742 of VHL gene and low pathological pT and between rs779805 of VHL gene and high pathological pT. As is generally known, tumor size, tumor grade, and pathologic pT are one of the best known factors with proven prognostic significance in kidney cancer. Therefore, although both investigated SNPs do not directly affect survival, they can be useful as prognostic biomarkers in ccRCC patients. To prove our observations and transfer them to clinical management, larger sample size is required in order to determine the exact power of correlations between these 2 genetic polymorphisms and renal cancer in the Central European population.

## Author contributions

**Conceptualization:** Magdalena Chrabańska, Nikola Szweda-Gandor.

**Data curation:** Magdalena Chrabańska, Nikola Szweda-Gandor.

**Formal analysis:** Magdalena Chrabańska, Nikola Szweda-Gandor.

**Funding acquisition:** Magdalena Chrabańska, Bogna Drozdzowska.

**Investigation:** Magdalena Chrabańska, Nikola Szweda-Gandor.

**Methodology:** Magdalena Chrabańska, Nikola Szweda-Gandor.

**Project administration:** Magdalena Chrabańska, Bogna Drozdzowska.

**Resources:** Magdalena Chrabańska, Nikola Szweda-Gandor.

**Supervision:** Bogna Drozdzowska.

**Validation:** Nikola Szweda-Gandor.

**Visualization:** Magdalena Chrabańska, Nikola Szweda-Gandor.

**Writing – original draft:** Magdalena Chrabańska.

**Writing – review & editing:** Nikola Szweda-Gandor, Bogna Drozdzowska.

## Supplementary Material


