# Supplementary material for: Does the VHL polymorphisms rs779805 and rs1642742 affect renal cell carcinoma susceptibility, prognosis and survival in Central European population?
Source: Medicine (Baltimore). 2023 Dec 15;102(50):e36540. doi: 10.1097/MD.0000000000036540 (PMC10727644; doi:10.1097/MD.0000000000036540)
Supplement: Supplementary file 1 [file medi-102-e36540-s001.docx]

**Does the VHL polymorphisms rs779805 and rs1642742 affect renal cell carcinoma susceptibility, prognosis and survival in Central European population?**

Magdalena Chrabańska^1^* MD, PhD, Nikola Szweda-Gandor^2^ PhD, Bogna Drozdzowska^1^ Professor

**NM_000551.4:c.*294G>A**

**Homo sapiens von Hippel-Lindau tumor suppressor (VHL), transcript variant 1, mRNA**

- **Selected genome build: GRCh38**
- **Map location: 3p25.3**
- **Transcript Flag: MANE Select**
- **CCDS ID:** [**CCDS2597.1**](https://www.ncbi.nlm.nih.gov/CCDS/CcdsBrowse.cgi?REQUEST=CCDS&DATA=CCDS2597.1)

#### HGVS-compliant variant descriptions

| **Type** | **Variant Description** | **Link to Reference sequence Record** |
| --- | --- | --- |
| Transcript (:c.) | [NM_000551.4:c.*294G>A](https://variantvalidator.org/service/validate/) | [NM_000551.4](https://www.ncbi.nlm.nih.gov/nuccore/NM_000551.4) |
| Protein (:p.) | [NP_000542.1:p.?](https://variantvalidator.org/service/validate/) | [NP_000542.1](https://www.ncbi.nlm.nih.gov/nuccore/NP_000542.1) |
| Protein (:p.) | [NP_000542.1:p.?](https://variantvalidator.org/service/validate/) | [NP_000542.1](https://www.ncbi.nlm.nih.gov/nuccore/NP_000542.1) |
| LRG Protein (:p.) | [LRG_322p1:p.?](https://variantvalidator.org/service/validate/) |  |
| LRG Protein (:p.) | [LRG_322p1:p.?](https://variantvalidator.org/service/validate/) |  |

##### Genomic Variants

| **Variant Description** | **VCF Description** | **Link to GenBank** |
| --- | --- | --- |
| [NC_000003.12:g.10150259G>A](https://variantvalidator.org/service/validate/) | GRCh38:3:10150259:G:A | [NC_000003.12](https://www.ncbi.nlm.nih.gov/nuccore/NC_000003.12) |
| [NC_000003.11:g.10191943G>A](https://variantvalidator.org/service/validate/) | GRCh37:3:10191943:G:A | [NC_000003.11](https://www.ncbi.nlm.nih.gov/nuccore/NC_000003.11) |

##### Variant Exon/Intron positions

| **Genomic/Gene accession** | **Exon/Intron at variant start** | **Exon/Intron at variant end** |
| --- | --- | --- |
| NC_000003.11 | 3 | 3 |
| NC_000003.12 | 3 | 3 |

##### Genomic descriptions

| **Reference Sequence Type** | **Variant Description** |
| --- | --- |
| Chromosomal GRCh37 | NC_000003.11:g.10191943G>A |
| Chromosomal GRCh38 | NC_000003.12:g.10150259G>A |

##### Transcript and protein descriptions

| **Reference Sequence Type** | **Variant Description** |
| --- | --- |
| Transcript | NM_000551.4:c.*294G>A |
| Protein single letter code | NP_000542.1:p.? |
| Protein three letter code | NP_000542.1:p.? |

rs 1642742 Context Sequence [VIC/FAM]GGACAGCTTGTATGTAAGGAGGTTT[A/G]TATAAGTAATTCAGTGGGAATTGCA

#### Gene Information

| **Attribute** | **Identifier** | **Source** |
| --- | --- | --- |
| Symbol | VHL | [HGNC](https://www.genenames.org/data/gene-symbol-report/#!/hgnc_id/HGNC:12687) |
| Name | von Hippel-Lindau tumor suppressor | [HGNC](https://www.genenames.org/data/gene-symbol-report/#!/hgnc_id/HGNC:12687) |
| HGNC ID | HGNC:12687 | [HGNC](https://www.genenames.org/data/gene-symbol-report/#!/hgnc_id/HGNC:12687) |
| NCBI Gene ID | 7428 | [NCBI](https://www.ncbi.nlm.nih.gov/gene/7428) |
| Ensembl Gene ID | ENSG00000134086 | [Ensembl](https://www.ensembl.org/Homo_sapiens/Gene/Summary?db=core;g=ENSG00000134086) |
| OMIM ID | 608537 | [OMIM](https://www.omim.org/entry/608537) |
| CCDS ID | CCDS2598 | [CCDS](https://www.ncbi.nlm.nih.gov/projects/CCDS/CcdsBrowse.cgi?REQUEST=ALLFIELDS&DATA=CCDS2598&ORGANISM=0&BUILDS=CURRENTBUILDS) |
| CCDS ID | CCDS2597 | [CCDS](https://www.ncbi.nlm.nih.gov/projects/CCDS/CcdsBrowse.cgi?REQUEST=ALLFIELDS&DATA=CCDS2597&ORGANISM=0&BUILDS=CURRENTBUILDS) |

**NM_000551.3:c.-195G>A**

**Homo sapiens von Hippel-Lindau tumor suppressor (VHL), transcript variant 1, mRNA**

- **Selected genome build: GRCh38**
- **Map location: 3p25.3**
- **Transcript Flag: RefSeq Select**
- **CCDS ID:** [**CCDS2597.1**](https://www.ncbi.nlm.nih.gov/CCDS/CcdsBrowse.cgi?REQUEST=CCDS&DATA=CCDS2597.1)

**Warnings output during validation**

- A more recent version of the selected reference sequence NM_000551.3 is available (NM_000551.4): NM_000551.4:c.-195G>A MUST be fully validated prior to use in reports: select_variants=NM_000551.4:c.-195G>A

**HGVS-compliant variant descriptions**

| **Type** | **Variant Description** | **Link to Reference sequence Record** |
| --- | --- | --- |
| Transcript (:c.) | [NM_000551.3:c.-195G>A](https://variantvalidator.org/service/validate/) | [NM_000551.3](https://www.ncbi.nlm.nih.gov/nuccore/NM_000551.3) |
| Transcript (:c.) | [LRG_322t1:c.-195G>A](https://variantvalidator.org/service/validate/) | [LRG_322](http://ftp.ebi.ac.uk/pub/databases/lrgex/LRG_322.xml) |
| RefSeq Gene (:g.) | [NG_008212.3:g.5019G>A](https://variantvalidator.org/service/validate/) | [NG_008212.3](https://www.ncbi.nlm.nih.gov/nuccore/NG_008212.3) |
| LRG (:g.) | [LRG_322:g.5019G>A](https://variantvalidator.org/service/validate/) | [LRG_322](http://ftp.ebi.ac.uk/pub/databases/lrgex/LRG_322.xml) |
| Protein (:p.) | [NP_000542.1:p.?](https://variantvalidator.org/service/validate/) | [NP_000542.1](https://www.ncbi.nlm.nih.gov/nuccore/NP_000542.1) |
| Protein (:p.) | [NP_000542.1:p.?](https://variantvalidator.org/service/validate/) | [NP_000542.1](https://www.ncbi.nlm.nih.gov/nuccore/NP_000542.1) |
| LRG Protein (:p.) | [LRG_322p1:p.?](https://variantvalidator.org/service/validate/) | [LRG_322](http://ftp.ebi.ac.uk/pub/databases/lrgex/LRG_322.xml) |
| LRG Protein (:p.) | [LRG_322p1:p.?](https://variantvalidator.org/service/validate/) | [LRG_322](http://ftp.ebi.ac.uk/pub/databases/lrgex/LRG_322.xml) |

**Genomic Variants**

| **Variant Description** | **VCF Description** | **Link to GenBank** |
| --- | --- | --- |
| [NC_000003.12:g.10141653G>A](https://variantvalidator.org/service/validate/) | GRCh38:3:10141653:G:A | [NC_000003.12](https://www.ncbi.nlm.nih.gov/nuccore/NC_000003.12) |
| [NC_000003.11:g.10183337G>A](https://variantvalidator.org/service/validate/) | GRCh37:3:10183337:G:A | [NC_000003.11](https://www.ncbi.nlm.nih.gov/nuccore/NC_000003.11) |

**Variant Exon/Intron positions**

| **Genomic/Gene accession** | **Exon/Intron at variant start** | **Exon/Intron at variant end** |
| --- | --- | --- |
| NC_000003.11 | 1 | 1 |
| NC_000003.12 | 1 | 1 |
| NG_008212.3 | 1 | 1 |

##### Genomic descriptions

| **Reference Sequence Type** | **Variant Description** |
| --- | --- |
| Gene | NG_008212.3:g.5019G>A |
| Chromosomal GRCh37 | NC_000003.11:g.10183337G>A |
| Chromosomal GRCh38 | NC_000003.12:g.10141653G>A |

##### Transcript and protein descriptions

| **Reference Sequence Type** | **Variant Description** |
| --- | --- |
| Transcript | NM_000551.3:c.-195G>A |
| Protein single letter code | NP_000542.1:p.? |
| Protein three letter code | NP_000542.1:p.? |

rs 779805 Context Sequence [VIC/FAM]GGCCTAGCCTCGCCTCCGTTACAAC[A/G]GCCTACGGTGCTGGAGGATCCTTCT.

#### Gene Information

| **Attribute** | **Identifier** | **Source** |
| --- | --- | --- |
| Symbol | VHL | [HGNC](https://www.genenames.org/data/gene-symbol-report/#!/hgnc_id/HGNC:12687) |
| Name | von Hippel-Lindau tumor suppressor | [HGNC](https://www.genenames.org/data/gene-symbol-report/#!/hgnc_id/HGNC:12687) |
| HGNC ID | HGNC:12687 | [HGNC](https://www.genenames.org/data/gene-symbol-report/#!/hgnc_id/HGNC:12687) |
| NCBI Gene ID | 7428 | [NCBI](https://www.ncbi.nlm.nih.gov/gene/7428) |
| Ensembl Gene ID | ENSG00000134086 | [Ensembl](https://www.ensembl.org/Homo_sapiens/Gene/Summary?db=core;g=ENSG00000134086) |
| OMIM ID | 608537 | [OMIM](https://www.omim.org/entry/608537) |
| CCDS ID | CCDS2598 | [CCDS](https://www.ncbi.nlm.nih.gov/projects/CCDS/CcdsBrowse.cgi?REQUEST=ALLFIELDS&DATA=CCDS2598&ORGANISM=0&BUILDS=CURRENTBUILDS) |
| CCDS ID | CCDS2597 | [CCDS](https://www.ncbi.nlm.nih.gov/projects/CCDS/CcdsBrowse.cgi?REQUEST=ALLFIELDS&DATA=CCDS2597&ORGANISM=0&BUILDS=CURRENTBUILDS) |
